# Acute myocardial infarction without significant coronary stenosis: evaluation by LE-CMR and CT coronary angiography

**DOI:** 10.1186/1532-429X-13-S1-P76

**Published:** 2011-02-02

**Authors:** Annachiara Aldrovandi, Daniele Arduini, Filippo Cademartiri, Chiara Martini, Diego Ardissino

**Affiliations:** 1 Department of Cardiology, Azienda Ospedaliero-Universitaria di Parma, Parma, Italy; 2 Department of Radiology, Azienda Ospedaliero-Universitaria di Parma, Parma, Italy

## Introduction

It is known that 9-31% of women and 4-14% of men with AMI have normal coronary arteries or non significant coronary disease at angiography. These patients represent a diagnostic and therapeutic challenge. Late gadolinium enhancement (LGE) cardiovascular magnetic resonance (CMR) imaging can detect and quantify myocardial scar. Multislice computed tomography can non invasively identify the presence of coronary plaques even in the absence of significant coronary artery stenosis.

## Purpose

This study evaluated the presence and characteristics of coronary atherosclerosis detected by 64-slice computed tomography angiography in patients with acute myocardial infarction (AMI) confirmed by late gadolinium-enhanced cardiac magnetic resonance but without significant coronary artery stenosis.

## Methods

Thirty-one consecutive patients with AMI but without significant coronary stenosis at coronary angiography underwent LGE-CMR and CTCA. Only patients with an area of myocardial infarction identified by LGE-CMR were included in the study. All coronary segments were assessed for the presence of coronary plaques

## Results

LGE-CMR showed an anterior myocardial infarction in 19 (61.3 %) patients, an inferior MI in 7 (22.5%) pts and a lateral MI in 5 (16.2%) pts. Twenty-four (5.1%) of the 447 coronary segments were not evaluable by CTCA. CTCA identified 56 coronary plaques and 36 (64.3%) plaques were located in infarct-related arteries (IRA) and 20 (35.7%) plaques in non infarct-related arteries. In IRA, 15 plaques were non calcified, 11 were mixed, and 10 were calcified; in non IRA, 15 plaques were calcified, 2 were non calcified, and 3 were mixed. The mean plaque area was significantly higher in IRA compared to non IRA plaques (6.9±6.6 mm^2^ vs 3.1±1.7 mm^2^, p=0.015) as well as the mean percent stenosis (36.1%±15 vs 25.5%±12, p=0.011).

## Conclusions

CTCA detected coronary atherosclerotic plaques in segments of non stenotic coronary arteries that are underestimated by CA and identified a different plaque type distribution between IRA and non IRA. CTCA and LGE-CMR may have an incremental diagnostic value for the diagnosis of AMI on an atherosclerosis basis in patients without significant coronary stenosis.

